# Delayed Aspiration of Cerebrospinal Fluid From a Thoracic Epidural Catheter After Difficult Placement: A Case Report

**DOI:** 10.7759/cureus.65519

**Published:** 2024-07-27

**Authors:** Daniel A Arnaut, Daniel Ohlhausen, Hamed Sadeghipour

**Affiliations:** 1 Anesthesiology and Critical Care, Saint Louis University School of Medicine, St. Louis, USA; 2 Anesthesiology and Critical Care, SSM Health Saint Louis University Hospital, St. Louis, USA

**Keywords:** epidural space (ep), neuraxial anaesthesia, thoracic spinal anaesthesia, epidural catheter displacement, post dural puncture headache, surgical pain, difficult epidural, accidental dural puncture, thoracic epidural anesthesia, epidural catheter migration

## Abstract

A 69-year-old female with Crohn’s disease was admitted for open ileocecectomy with lysis of adhesions. The plan was to proceed with general endotracheal anesthesia and a thoracic epidural catheter for perioperative analgesia. Epidural access was attempted at the T10-11 and T11-12 interspaces, both of which resulted in accidental dural punctures. On the third attempt, the epidural catheter was inserted at the T9-10 interspace. Both the aspiration and test dose were negative. Thirty minutes later, after induction of general anesthesia, the catheter was again aspirated before the epidural pump was connected. Freely flowing, glucose-positive fluid was obtained, and the catheter was removed for the patient’s safety. This case suggests that accidental dural puncture may be a risk factor for inappropriate communication with the subarachnoid space. This can be assumed to increase the risk of unanticipated high or total spinal block and its life-threatening sequelae.

## Introduction

Aspiration of epidural catheters after placement is a common method used to rule out intrathecal and intravascular placement [1]. Aspiration is considered negative when fluid, whether it be blood or cerebrospinal fluid (CSF), cannot be aspirated freely from the catheter. In an awake, cooperative patient, negative aspiration is typically followed by a test dose of lidocaine with epinephrine [2]. A test dose is considered negative when the patient denies symptoms of intravascular local anesthetic injection (perioral paresthesia, tinnitus, metallic taste), there are no signs of intravascular epinephrine injection (increases in blood pressure and heart rate), and there are no signs of intrathecal local anesthetic injection (sudden weakness, slurred speech, unexpectedly high sensory block) [2,3].

Epidural catheters have the possibility of a rare but serious complication of migration [4-6]. Catheter migration can lead to intravascular, subdural, or subarachnoid cannulation. A catheter that has migrated to the subarachnoid space can be diagnosed by aspiration of CSF from the catheter. However, migration of the catheter can occur after initial negative aspiration and test dose and therefore can go unnoticed [4,5]. This can lead to potentially life-threatening complications due to high doses of local anesthetic being placed in the subarachnoid space, leading to high or total spinal block [7,8].

Usually, when freely flowing CSF is aspirated from the catheter, it is assumed to be intrathecal [1]. However, CSF can sometimes be aspirated from the epidural space, especially if there is a defect in the dura [1,9]. This is a poorly described phenomenon and can lead the anesthesiologist to misinterpret the position of an epidural catheter. There are no universal guidelines for troubleshooting the catheter in such a situation.

In our case, a difficult thoracic epidural catheter placement after two accidental dural punctures (ADPs) was thought to be successful due to negative aspiration and test dose. However, repeat aspiration half an hour later was positive for freely flowing, glucose-positive fluid. The anesthesia team had no way to safely delineate the position of the catheter without delaying the case, and the catheter was removed for the patient’s safety. This case highlights the diagnostic ambiguity of a delayed positive epidural catheter aspiration test, as well as the need for guidelines in such circumstances.

## Case presentation

A 69-year-old female, with American Society of Anesthesiologists (ASA) class II, body mass index (BMI) of 21.47 kg/m2, and Crohn’s disease, was admitted for open ileocecectomy with lysis of adhesions. She had a remote history of ileocecal resection complicated by anastomotic fibrotic stricture and chronic abdominal pain. Other medical history included gastroesophageal reflux disease (GERD), hiatal hernia, and glaucoma. The plan was to proceed with general endotracheal anesthesia with a thoracic epidural catheter placed preoperatively to help manage perioperative pain.

In the preoperative holding area, consent was obtained for thoracic epidural placement, and the pre-procedure timeout was completed. The patient was connected to standard ASA monitors and received 25 mcg of fentanyl and 1 mg of midazolam. With the patient in a sitting position, the skin was anesthetized with 3 cc 1% lidocaine. Using an 18G, 9 cm Tuohy needle (Braun Medical, Bethlehem, PA) and the loss of resistance technique with normal saline, the epidural was attempted using a midline approach. The sterile technique was used throughout the procedure. Loss of resistance occurred at a needle insertion depth of 4 cm. The first two attempts at the T10-11 and T11-12 interspaces were unsuccessful and resulted in a freely flowing CSF return through the needle hub. No blood loss or paresthesia were noted at any time.

On the third attempt, a 20G closed-tip epidural catheter (Braun Medical, Bethlehem, PA) was inserted at the T9-10 interspace to a length of 9 cm at the skin. Aspiration was negative at this time. A test dose injection of 3 cc lidocaine 1.5% with 1:200,000 epinephrine was administered through the catheter, which did not elicit a subjective response from the patient. She denied tinnitus, perioral paresthesia, metallic taste, and weakness of the lower extremities. Objectively, no changes in her vital signs were noted, including blood pressure and heart rate. The catheter was then secured at a length of 9 cm at the skin. Mastisol (Eloquest Healthcare, Ferndale, MI) was applied to the skin around the insertion site and catheter, followed by a sterile transparent dressing, thus preventing any manipulation of the catheter’s position by traction outside of the body. The edges of the dressing were also reinforced with silk tape. The patient was moved back into a supine position with the head of the bed at 30 degrees.

Thirty minutes later, the patient was transferred from the preoperative area to the operating room (OR). The anesthesia team that would be present for the operation was made aware that there had been difficulty with placing the epidural but that the initial test dose had been negative. General anesthesia was achieved without any issues. The epidural catheter was then reassessed before attaching the epidural pump, which revealed catheter position was still 9 cm at the skin. However, aspiration of the catheter yielded a clear liquid, which flowed freely through the catheter. Aspiration was halted after 2 cc’s were easily obtained. Due to concern for possible unintentional high or total spinal block, the decision was made to remove the catheter. The tip was confirmed to be intact upon removal. The patient underwent surgery without complications. This fluid was sent to the lab and was later confirmed to be CSF by an elevated glucose level of 64 mg/dL.

Following extubation, she was moved to the post-anesthesia care unit (PACU) and recovered from anesthesia without any issues. Her pain was managed by IV opioids as needed, together with bilateral transversus abdominis plane (TAP) blocks, using 20 mL of bupivacaine 0.25% and 4 mg dexamethasone injected on each side. She tolerated the blocks well. Postoperatively, her pain was managed with oral opioids as needed and adjuncts. Of note, the patient developed a mild diffuse headache and subjective bilateral hearing loss postoperatively, which we attributed to the dural punctures. The headache and hearing loss resolved within 48 hours, and there were no residual neurologic symptoms.

## Discussion

ADP is a common complication of epidural catheter placement, occurring in 0.4-6.0% of patients [10]. Dural puncture is often diagnosed by the appearance of clear fluid flowing from the needle hub when advancing the needle. Factors that place patients at higher risk of ADPs in the thoracic spine are not well studied but are thought to include conditions that distort the normal curvature of the spine, such as scoliosis. It has also been shown that the incidence of ADPs is highest during repeated attempts for epidural placement [10].

Once an ADP occurs, there are three standard options: (1) place the catheter intrathecally; (2) attempt epidural access at a different intervertebral space; or (3) elect to forego neuraxial anesthesia entirely [11]. Placing an epidural catheter into the intrathecal space after ADP has several purported benefits: rapid initiation of analgesia; the avoidance of need for further attempts to achieve epidural analgesia and possible repeat accidental dural puncture; and the potential reduction of post-dural puncture headache (PDPH) [7,10,12]. The bulk of the literature supporting these claims comes from studies on lumbar epidurals for obstetric patients. There is limited literature describing the placement of intrathecal catheters following ADP in the thoracic spine [13]. In our case, the risk of high or total spinal block and its associated complications outweighed the benefits of thoracic spinal anesthesia. Moreover, our institution does not have protocols in place to manage thoracic spinal catheters. Therefore, we elected to re-site the epidural catheter at another intervertebral level.

Following ADP, the incidence of PDPH could be as high as 76-85% [14]. Symptoms of PDPH include headaches typically focused in the frontal-occipital distribution, worsened by sitting or standing and alleviated by lying down. Additional symptoms include neck stiffness, tinnitus, hypoacusia, photophobia, and nausea. The mechanism of the condition is suspected to be due to reduced CSF pressure due to leakage of CSF from the intrathecal space [14]. As in our patient, these symptoms typically resolve within a week. Our patient’s development of PDPH supports the notion that there was a significant leak of CSF into the epidural space.

In our case, initial aspiration and test dose were negative, but freely flowing CSF was aspirated in the OR only half an hour later. There are several distinct possibilities that may explain this phenomenon. These possibilities include the following: (1) catheter tip migration from the epidural or subdural space into the subarachnoid space; (2) intrathecal placement with initial false negative aspiration and a false negative test dose; (3) filling of the epidural space with CSF, which surrounded the catheter; or (4) the catheter tip migrating to lie adjacent to a dural defect within the epidural space.

Epidural catheter migration into intravascular, subdural, or subarachnoid space is a rare complication of epidural anesthesia. Epidural catheter migration is difficult to detect because of a wide range of potential symptoms and a lack of diagnostic guidelines. If our epidural catheter did, in fact, migrate into the subarachnoid space, intrathecal injection of epidural-dose local anesthetic and narcotic could have resulted in respiratory arrest and cardiovascular collapse due to a high or total spinal block [6-8].

If the catheter was placed into the intrathecal space initially, it is possible that the lack of response to the test dose was a false negative. However, with our standardized follow-up questions after administering the test dose and the patient's ability to raise her legs to get back into bed, the sensitivity approaches 100%. Therefore, the test dose is excellent for ruling out intrathecal placement [3]. On the other hand, the test dose has been criticized for its poor specificity, resulting in many false positives [2]. This is a topic for another discussion. In our case, the aspiration test preceding the test dose was also negative; this may have been due to tissue blocking flow through the catheter, or the catheter was not intrathecal. To our knowledge, there is no data regarding the sensitivity of aspiration for detecting intrathecal cannulation. However, in obstetric patients, the rate of accidental intrathecal injection after negative aspiration is rare, estimated to occur in 0.0008-0.06% of patients [2]. Both the sensitivity of the test dose and the rarity of intrathecal cannulation after negative aspiration led us to believe that our catheter was not initially placed into the intrathecal space.

Another possibility is that the catheter was seated properly in the epidural space, hence the initial negative aspiration. Due to the multiple ADPs, the epidural space filled with CSF as it leaked from the subarachnoid space, opening the epidural space around the catheter. This could theoretically lead to a positive subsequent aspiration test [15]. Similarly, if the catheter tip migrated to overlie the dural defects, aspiration may pull fluid directly from the intrathecal space through these defects. The minimal resistance to flow observed would suggest a significant leak, and administration of anesthetic through this catheter could theoretically result in spinal anesthesia by diffusion of epidural anesthetic into the intrathecal space through dural defects.

There are only a few articles describing the aspiration of CSF from the epidural space [1,9,15]. One case series describes positive aspiration from epidural catheters after dural puncture for combined spinal-epidural (CSE) anesthesia, where 0.75 cc of freely flowing CSF was aspirated in two cases. Postoperatively, the anesthesia providers cautiously administered spinal-dose anesthetic into the catheters and titrated to effect. To achieve adequate analgesia, they required epidural doses of anesthetic. In short, the catheters were successfully used for epidural anesthesia despite the positive aspiration of CSF. Later, the catheter positions were radiographically confirmed to be in the epidural space, demonstrating that CSF can be aspirated from a properly seated epidural catheter after a dural puncture [9]. The CSE cases differ from ours because their aspiration attempt was positive on initial evaluation of the catheter, and the dural puncture was intentional and with a smaller gauge needle. Additionally, their epidural catheters were placed in the lumbar spine.

Another relevant case report described failed spinal anesthesia after multiple attempts to place an epidural catheter [15]. In their case, epidural catheter placement was aborted after two ADPs. Spinal anesthesia was then attempted as an alternative but failed despite the robust return of CSF through the spinal needle prior to administration of anesthetic. The authors hypothesized that their spinal anesthetic may have failed for two reasons: either the anesthetic was administered intrathecally and leaked out into the epidural space through the dural defects from ADPs, or the spinal anesthetic was administered into the epidural space, which they mistook for the subarachnoid space due to return of CSF [15]. The latter hypothesis supports the notion that ADPs can result in significant leakage of CSF into the epidural space.

A CT myelogram with administration of contrast through the catheter can be utilized to delineate the location of the catheter tip [16]. This was not a viable option for our case as the patient’s surgery would have needed to be canceled due to time limitations. A case report with a similar clinical scenario as our case described the use of ultrasound pulsed-wave Doppler to localize an epidural catheter tip [17]. This is a promising technique, but more research is required to determine its utility. Without radiographic evidence, an epidural catheter with positive aspiration of glucose-positive fluid should be treated as intrathecal until proven otherwise.

Theoretically, if our patient were awake, we could have attempted to administer spinal-dose anesthetic through our epidural catheter and monitor for a response, titrating to effect [8,9]. However, our patient was already under general anesthesia when positive aspiration was discovered. As mentioned above, our institution does not have protocols in place for managing thoracic spinal catheters. Because continuous spinal anesthesia is not commonplace at our institution, we felt that the risk of a drug dose error was too great if we left the catheter intrathecally. Had epidural-dose anesthetic been delivered intrathecally in our frail patient, cardio-respiratory arrest would likely have occurred among other catastrophic sequelae [7].

## Conclusions

In our case, we postulate that multiple dural defects from ADPs may have caused significant leakage of CSF into the epidural space, resulting in positive aspiration of CSF from the catheter. Alternatively, epidural catheter migration may have occurred due to a change in patient position, diverting the epidural catheter over or into a defect in the dura from the previous insertion attempts. Finally, the catheter may have been initially inserted into the intrathecal space, and both aspiration and test dose were negative for an unknown reason. Regardless of the cause, we elected to remove the catheter due to uncertainty of its positioning, a lack of institutional protocols for managing thoracic spinal catheters, and concern that a drug dose error may occur if the catheter was left in place. Our case suggests that ADPs may be a risk factor for inappropriate communication with the subarachnoid space, which may not be apparent in the initial evaluation of the epidural catheter. This can be assumed to increase the risk of unanticipated high or total spinal block and its life-threatening sequelae.
